# Supplementary figures and images for: Investigating passive eDNA samplers and submergence times for marine surveillance
Source: PeerJ. 2025 Mar 6;13:e19043. doi: 10.7717/peerj.19043 (PMC11890302; doi:10.7717/peerj.19043)

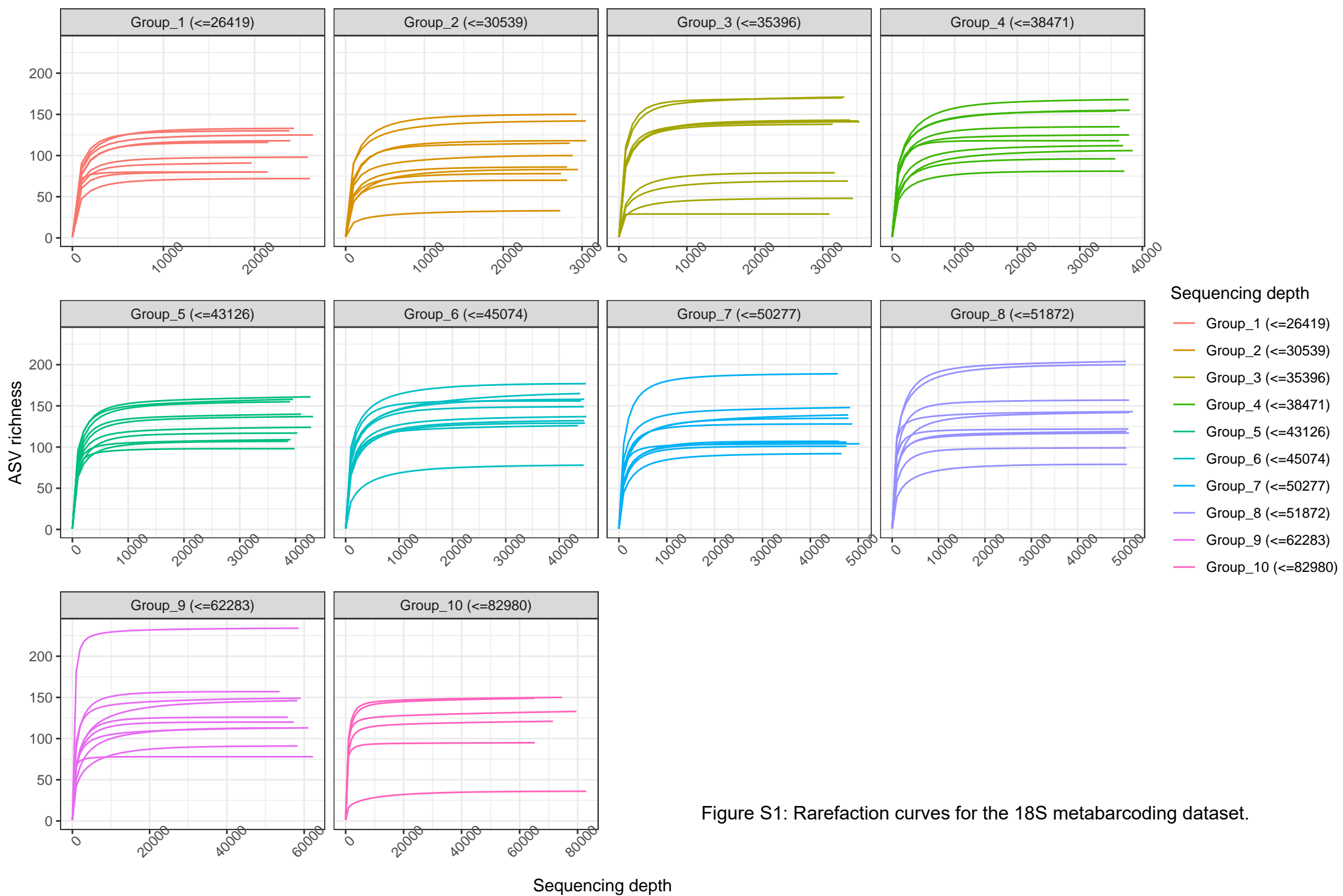

Supplement: Supplemental Information 1 [file peerj-13-19043-s001.pdf]
