# Supplementary material for: Investigating passive eDNA samplers and submergence times for marine surveillance
Source: PeerJ. 2025 Mar 6;13:e19043. doi: 10.7717/peerj.19043 (PMC11890302; doi:10.7717/peerj.19043)

Figure S2: Principal component analysis(PCA) of community diversity matrix with deployment time displayed as an ordisurf layer.

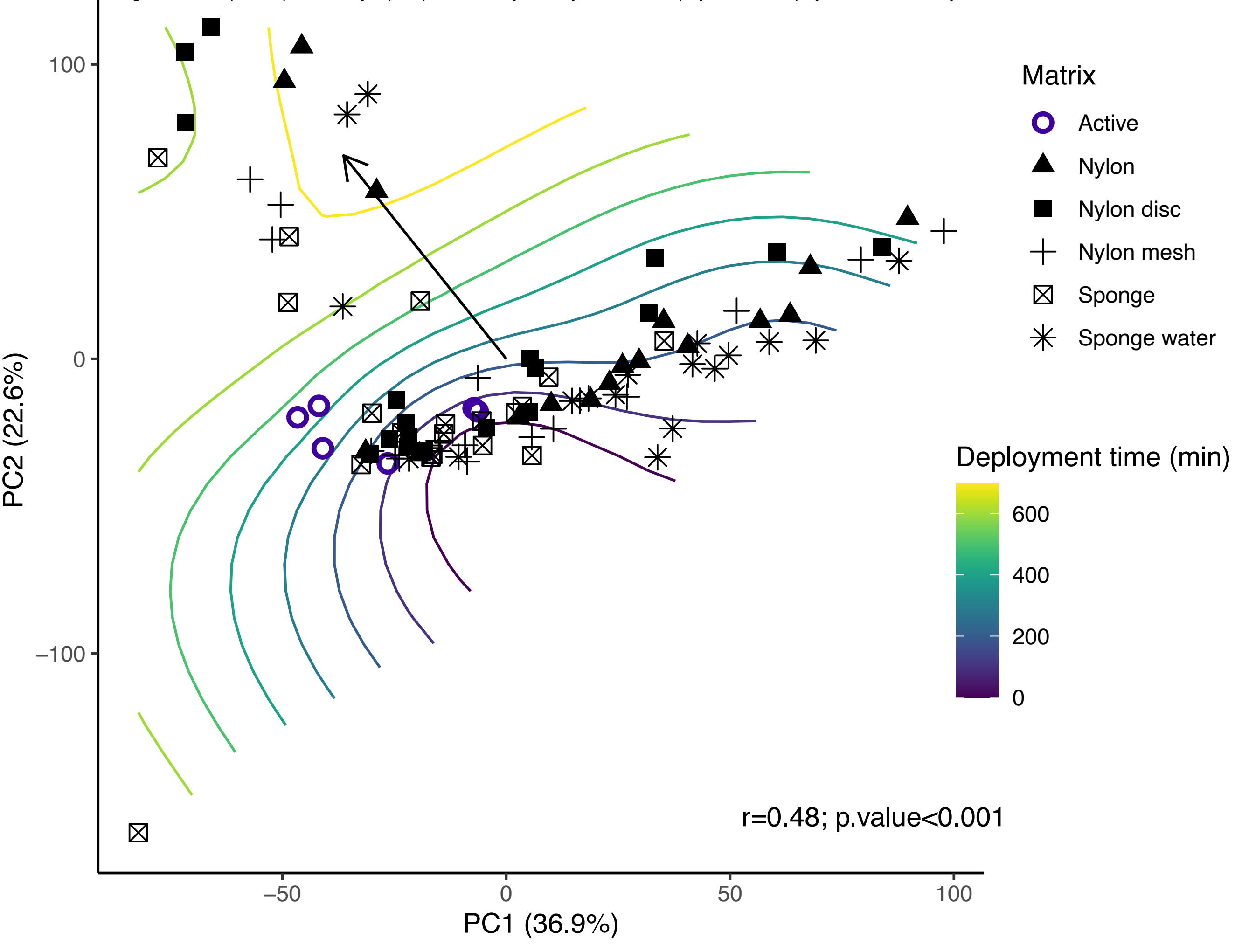

Supplement: Supplemental Information 2 — In addition, the correlation of deployment time with biological assemblages is showed with an arrow, with Pearson (r) and p-value displayed in the lower right corner. [file peerj-13-19043-s002.pdf]
