# Supplementary material for: Investigating passive eDNA samplers and submergence times for marine surveillance
Source: PeerJ. 2025 Mar 6;13:e19043. doi: 10.7717/peerj.19043 (PMC11890302; doi:10.7717/peerj.19043)
Supplement: Supplemental Information 5 [file peerj-13-19043-s005.docx]

Table S3: Permutational analysis of variance testing the effect of deployment time per matrix on the eukaryotic community (18S rRNA).

| Matrix | Term | df | R^2^ | *p.*value |  |
| --- | --- | --- | --- | --- | --- |
| Nylon | Time | 1 | 0.38 | **0.001** |  |
|  | Residual | 15 | 0.62 |  |  |
| Nylon disc | Time | 1 | 0.44 | **0.001** |  |
|  | Residual | 16 | 0.56 |  |  |
| Nylon mesh | Time | 1 | 0.44 | **0.001** |  |
|  | Residual | 16 | 0.56 |  |  |
| Sponge | Time | 1 | 0.17 | **0.003** |  |
|  | Residual | 17 | 0.070.83 |  |  |
| Sponge water | Time | 1 | 0.36 | **0.001** |  |
|  | Residual | 16 | 0.64 |  |  |
